# All-digital training course in neurophysiology: lessons learned from the COVID-19 pandemic

**DOI:** 10.1186/s12909-021-03062-3

**Published:** 2022-01-03

**Authors:** Michael Duszenko, Nicole Fröhlich, Ariane Kaupp, Olga Garaschuk

**Affiliations:** Department of Neurophysiology, Eberhard-Karls University, Keplerstr. 15, 72074 Tübingen, Germany

**Keywords:** COVID-19 pandemic, physiology, all-digital training course, laboratory, remote, virtual

## Abstract

**Background:**

The social distancing and suspension of on-campus learning, imposed by the COVID-19 pandemic, are likely to influence medical training for months if not years. Thus, there is a need for digital replacement for classroom teaching, especially for hands-on courses, during which social distancing is hardly possible. Here, we investigated students’ learning experience with a newly designed digital training course in neurophysiology, with intercalated teaching blocks in either asynchronous (unsupervised online lectures and e-labs) or synchronous (online seminars, supervised by instructors) formats.

**Methods:**

The accompanying anonymized prospective study included 146 student participants. At the beginning and the end of the course, students were invited to answer anonymous online questionnaires with 18 and 25 items, respectively. We conducted both qualitative analyses of students’ survey responses and statistical analyses of the results of cohort-specific summative examinations. The summative assessment results were compared both between 4 current cohorts and with the respective historical cohorts.

**Results:**

Despite having little prior experience with e-learning (4.5 on the 1-7 scale), students adapted remarkably well to this online format. They appreciated its higher flexibility, time efficiency, student-oriented nature (especially when using inverted classroom settings), tolerance towards the individual learning style and family circumstances, and valued the ability to work through lectures and e-labs at their own learning speed. The major complaints concerned diminished social contacts with instructors and fellow students, the inability to ask questions as they occur, and the lack of sufficient technical expertise. The students valued the newly developed e-labs, especially the implementation of interactive preparative measures (PreLabs) and the intuitive lab design offered by the chosen software (*Lt Platform* from AD Instruments). The summative examinations at the end of the course documented the quality of knowledge transfer, which was comparable to that of previous classically instructed cohorts.

**Conclusion:**

Despite the missing personal contact between the faculty and the students, inherent to online teaching, the all-digital training course described here proofed to be of good educational value and, in case the pandemic continues, is worse considering for the future. Some of the described building blocks, like digital lectures or interactive PreLabs, may survive the pandemics to enrich the medical education toolbox in the future.

## Background

COVID-19 pandemic is not completely controlled yet, and the worldwide scientific community continues to identify new, even more infectious virus mutants [1]. Thus, despite the availability of vaccines, possible lockdowns, the suspension of in-person learning in classrooms, and social distancing may influence medical training for months if not years. Many students are concerned as to how they will complete their practical courses. A recent survey by the American Physiological Society revealed that 58% of trainee respondents said that closures due to the COVID-19 pandemic may increase the time it takes them to complete their training [2]. For medical teachers, this development raises the demand to provide e-teaching with as little loss of teaching quality as possible [3, 4]. Important questions arising in this context and addressed by the current study include “how to motivate/engage students”, “how to ensure structured learning and proper knowledge transfer”, “under which circumstances are the positive effects of in-person laboratory experiences (e.g., reinforcement and deepening of lecture theory, development of transferable skills and knowledge of the relevant measurement techniques, experiences from working as a team, etc.) transferable to online courses”? Moreover, it is important to explore which novel teaching modalities, emerging as an acute response to the COVID-19 pandemic, should be permanently anchored in future curricula [4].

Before pandemic, all learning activities for preclinical medical students (3^rd^ and 4^th^ semester), dentists, and students of B.Sc. Molecular medicine on our campus were delivered in face-to-face mode. Due to the lockdown in spring 2020, we, like many other colleagues worldwide, were forced to develop the all-digital training course in neurophysiology (see below). To evaluate this concept and to understand students’ perceptions of the new education environment [5], we developed and performed among the students an anonymized study on a voluntary basis. The overall goal of this study was to obtain a qualitative and in parts quantitative assessment of the newly developed course and to find out whether our new concept meets the needs of the students, thus enabling sustainable and quality-assured development of the subject. The following objectives were of crucial importance: validation of the newly developed online formats by students and teachers in terms of study ability, effectiveness, quality assurance, and sustainability; comparison with the previously practiced attendance formats (for those students, who were studying physiology in winter semester 2019/2020); collection of cohort-specific quantitative data by means of summative testing. Based on the statistical evaluation of all collected data, we examined to what extent the newly developed e-learning concept gets acceptance of students/teachers; how well our students cope with such learning formats; to what extent a quality-assured transfer of knowledge is possible under pure e-learning conditions; and which learning formats can be kept after the COVID-19 pandemic thus promoting sustainable, evidence-based digitization of the teaching in physiology.

Since the 4^th^-semester medical students went straight into their first medical exam (Physikum), their respective performance data, as compared with the results of former years, are available. The first medical exam consists of a generalized written examination, centrally organized by the Institute for Medical and Pharmaceutical Exams and mandatory for all medical students in Germany, as well as an oral exam, locally performed at each university, where most of the teachers of our course were also examiners. Whereas the content-related data are specific for the teaching of neurophysiology, general data about the experience with and the acceptance of the e-learning modules likely equally applies to the other disciplines taught during preclinical medical training.

## Methods

### Ethical approval

Ethics approval was obtained from The Ethics Committee at the Medical Faculty of the Eberhard-Karls-University and the University Hospital Tübingen ref. number 437/2020BO.

### Research design

An anonymized prospective study was conducted between the 1st of June and 31st of July 2020 amongst undergraduate students in medicine (3rd and 4th semester); dentistry (4th and 5th semester), and B.Sc. Molecular medicine at the Medical faculty of the University of Tübingen.

### Sampling

All 425 students, admitted to the neurophysiology course in the summer semester of 2020, were invited to participate in the study. The invitation was posted within the protected area on the Ilias platform, used by all students to access the teaching materials, and repeated by the Study officer of the Department of Neurophysiology during the first introductory lecture. Those interested to participate were free to click on the link below the invitation, which brought them to the participant information sheet and a detailed consent form. All study participants provided voluntary written consent to participate in the study.

Data were sampled using two anonymous online questionnaires with 18 and 25 items, respectively. The first questionnaire was filled at the beginning and the second follow-up survey - at the end of the course. The students were asked to provide quantitative estimations using, depending on the question, one of the two different Likert scales: the 1-7 scale from 1 (very experienced) to 7 (no experience) and the scale from 1 (very good) to 5 (bad), comparable to the school grading system. In the first questionnaire, students were asked about sociodemographic items (e.g., gender, age, study subject, and education history), available technical equipment (e.g., PC, laptop, tablet, or smartphone), their routine use of e-learning contents and previous experiences with synchronous and asynchronous online-based teaching formats as well as platforms used for online teaching. The latter included e-learning platforms ILIAS and moodle, used by the University of Tübingen, commercial online platforms AMBOSS and via medici, helping students to prepare for the written state exam, as well as Lt Platform from AD Instruments, used for e-laboratories. In the second questionnaire, the participants were asked to comment on their experience with each course module (i.e., lectures, e-laboratories, organ centered and integrated seminars) from the technical and educational points of view as well as regarding the relevance of knowledge obtained for the forthcoming state exam. Before the pandemic, some participants studied cardiopulmonary and exercise physiology on campus. These participants were invited to compare the face-to-face and e-learning concepts in teaching physiology regarding their learning success. Finally, the performance of the student cohorts in the institutional neurophysiology exam as well as in the written part of the Germany-wide first medical exam was compared with the students’ performance in previous years to draw conclusions about the quality of knowledge transfer in the e-learning settings.

### Data analyses and statistics

The thematic analysis of the data was approached from the exploratory point of view, aiming to gain insights into participants’ experiences and needs [6]. We applied the grounded theory approach [7, 8], using the following data analysis algorithm: initial coding was performed independently by three different researchers. This involved immersion into the data, note-making, identification of key points as initial codes, and collapsing the codes into themes arising from the dataset. Subsequently, two senior colleagues reviewed the initial coding, wrote informal analytic notes about the dataset, identified new codes, and generated conceptual categories according to the meanings and interrelationships identified. The same persons tested whether the results of this process seemed to conflict with the initial phenomenological analysis and coding, and reviewed codes and categories for errors and omissions. During this analysis step, we did not identify additional categories and ensured that the categories are sufficiently explained.

When analyzing the quantitative data, all statistical tests were two-sided. The even distribution of the data was tested using the Shapiro-Wilk test, while the Kruskal-Wallis test was used for comparing more than two not evenly distributed data sets. P values <0.05 were considered significant. Unless otherwise indicated, data are presented as median ± interquartile range (IQR).

## Results

This anonymized prospective study included 146 participants out of the 425 students admitted to the course and was conducted as a part of the online teaching of neurophysiology in the summer semester of 2020. The participants belonged to one of the five cohorts of students: medicine (3rd and 4th semester); dentistry (4th and 5th semester) and B.Sc. Molecular medicine. Before the pandemic, 68 out of 146 participants studied cardiopulmonary and exercise physiology on campus. These participants were invited to compare the face-to-face and e-learning concepts in teaching physiology regarding their learning success.

The survey results obtained are representative of undergraduate medical students, as 34,6% of students participated in this study. Because of the lower degree of participation among students of dentistry (19.4%) and B.Sc. Molecular medicine (11.5%), no representative survey data could be obtained for these two cohorts.

### Participant characteristics

According to the data from the first questionnaire, out of all study participants, 88% were medical students, equally distributed between the 3^rd^ and the 4^th^ semesters, 10% were students of dentistry, and 2% studied molecular medicine. 73% of participants were females, consistent with the general gender distribution among our students, of which 70% are females. Nearly half of the cohort (49%) started their study right after high school, 44% of the students completed vocational training before the study and 7% even obtained a university degree. For the remote training 47% of participants used personal computers or laptops, 27% used a tablet computer, and 21% a smartphone. 5% of students did not possess their own device and shared either a PC/laptop or tablet with others. At least 12% of our students used macOS operating system.

When asked, how familiar they are with supervised and unsupervised online teaching formats (1-7 scale), the degree of familiarity among students was moderate: 4.5 for the unsupervised formats and 4.4 for supervised courses. As to online platforms, 29% of students were familiar with ILIAS, 26% with AMBOSS, 21% with Lt Platform, 18% with *via medici* from Thieme, and only 1% with *moodle*. Rating their grade of experience on the abovementioned 1-7 scale, the values were 1.7 (ILIAS), 2.4 (AMBOSS), 4.0 (Lt Platform and *via medici*), and 6.7 (moodle). Using grades from 1 (very good) to 5 (bad), the students ranked the overall quality of the different platforms as follows: AMBOSS (1.7), *via medici* (1.9), Lt Platform (2.3), and ILIAS (2.4). As for available textbooks, 31% used e-books, 30% borrowed textbooks from the university’s library, 25% bought print versions, 11% used remote access to other libraries and 3% used other options. Finally, when asked to estimate their familiarity with programs to be used during the course (1-7 scale), the students were familiar with ILIAS (1.8), Microsoft office (1.9), and Zoom/Webex/DFNconf (2.2). The familiarity with the *Lt Platform* (2.6) was rather limited.

### Changes in learning behavior during the COVID-19 pandemic

We also asked whether and if yes how the learning habits of our students changed due to the COVID-19 pandemic. Due to the pandemic, 70% of the students have adapted their learning behavior; 53% of students studied alone, 22% studied with just one fellow student (tandem), 19% studied in small learning groups (up to 5 people), and 5% used digital formats like WhatsApp, Skype, Zoom, etc. to interact with other course participants; 2% studied together with a person from another course. When learning in small learning groups or with tandem partners, 50% of the participants met less than once a week, 32% met 1-2 times a week, 12% met 3-4 times a week and only 6% had daily meetings.

On average, the students invested 2.78 hours (h) into online teaching (in a supervised or unsupervised format) and additional 4.17 h into autodidactic learning. Based on their experience prior to our course, about half of the cohort (54%) preferred the traditional classroom teaching, while 22% favored the digital format. 24% of the students got along equally well with both formats. In addition, 70 students commented on their preference for the traditional teaching in free text. The main reasons mentioned by 61% of responders included the ability to communicate with their classmates, to directly ask questions to the tutor/lecturer as well as structured daily routine with set times for different courses (guidance), allowing the students to focus on one aspect and to be more effective overall. The changeover to e-learning was difficult for these students, as it forced prolonged sessions in front of a computer and required more effort, time, and personal responsibility, which rendered preparation for exams more difficult. The students also experienced some technical problems, with difficult or delayed access to general information and teaching materials. However, 29 students provided positive comments about e-learning, especially stressing the possibility to pause, repeat and rewind asynchronous presentations, flexibly structure their day, save traveling time, and individually adjust their learning rhythm, thus increasing learning efficiency.

Overall, most students had ambivalent experiences with digital teaching. On the one hand, the general campus atmosphere was lacking. On the other hand, the individualized time management, lack of stress, and flexibility of the day structure were valued by many students. The students appreciated the digitalization efforts made by the university, forced by the COVID-19 pandemic. The abrupt implementation of digital formats was rated satisfactory or better than expected.

### Description of the new all-digital teaching concept

This teaching intervention took place in form of an 8-week-long online neurophysiology teaching module in June-July 2020. Usually, during the module, the students can dedicate almost the entire time to studying neurophysiology. However, because of the COVID-19 pandemic, this cohort of students had to pass the written/oral tests in anatomy and biochemistry, which could not take place during the spring lockdown.

**Fig. 1:**
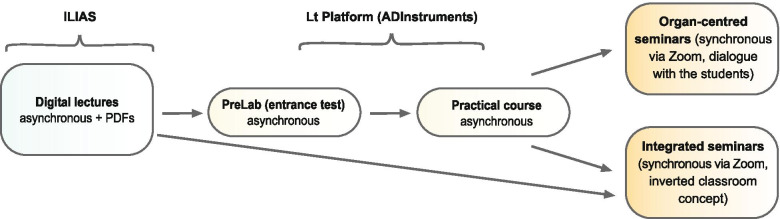
The flow chart illustrating our teaching concept. The knowledge acquisition begins with digital lectures. The arrows reflect the sequential buildup of knowledge and skills during the course

At our university, the face-to-face neurophysiology teaching block contains lectures (60 academic hours (a.h.)), a practical course (24 a.h.), an organ centered seminar (30 a.h.), and an integrated seminar (24 a.h.), compulsory for preclinical medical training in Germany. We followed this pre-set curriculum, transforming all parts into digital format. A hybrid teaching concept, with contents presented either in an unsupervised asynchronous (lectures, practical course) or supervised synchronous (seminars) way, was used (Fig. 1). Aiming to give the students a time guide and orientation [9, 10], we sequentially structured our asynchronous online materials, with some of the elements appearing and others disappearing after a given time. Thus, a new lecture appeared every day and stayed online, while each of the 6 e-labs was removed from the internet platform after one week. For the synchronous parts (Zoom meetings), defined cohorts of students (~ 25 people) were invited to participate at a fixed date and time. In the all-digital neurophysiology module offered, all teaching formats were interlocked, so that the next lesson required the knowledge/skills acquired during the previous lesson.

The daily lectures did not require any prior knowledge of neurophysiology. They aimed at providing the background and overview as well as at describing the topic in sufficient detail to prepare the students for the subsequent seminars and practical courses. Developing the asynchronous online practical course, consisting of 6 different e-laboratories, turned out to be the most challenging. To assure that students possess the knowledge required for the given practical course, we designed a PreLab training including initial tests, to be performed right before each practical course. In our concept, a good comprehension of respective lecture topics is a prerequisite for entering the subsequent practical course, providing praxis-oriented knowledge and skills (Fig. 1). In neurophysiology, the thematic focus of the course is centered on such organs/topics as nerve, muscle, sensation, vision, hearing, and higher brain functions. The course teaches several medical diagnostic tests/approaches including measurements of nerve conduction velocity, electroencephalography (EEG), electromyography (EMG), electrooculography (EOG), visual acuity, audiometry, etc. Usually, the experiments are carried out on campus in specially designated practical training rooms under the supervision of tutors. Each student spends on-site 4 a.h. per week, mounting in 6 weeks to a total of 24 a.h. Prior to each course, the students work through a practical guide describing in detail each experiment. To design the digital practical course, we tested several available platforms and finally chose the Lt platform from ADInstruments (https://www.adinstruments.com/) because of the following reasons: well-designed and easy-to-use software, availability of pre-designed modules in English and German languages, possibility to connect and disconnect hardware and thus allowing easy switching between the on-campus and online modules and acceptance by a large user community. To enable the students to work with the Lt Platform, we prepared an introduction video, which was available throughout the course.

Each but one of 6 e-laboratories included 3 different lessons. The PreLabs defined the relevant lecture/handbook topics, provided a concise recapitulation of the theoretical background including the practical guide, and assessed the student’s ability to apply the knowledge already obtained. For assessment, out of different question types supported by the platform, we mostly used single-choice questions with 5 answer options (this question type is also used during our written exam and the subsequent written state exam). Occasionally, we also used true/false, drag and drop, and ordering questions. Students answered and submitted all questions online to be scored and saved by the system and received the correct answers right afterward. While answering all PreLab questions was mandatory and a prerequisite for the continuation of the respective lesson, the scores achieved by the students served only for their own formative feedback. The teachers obtained the information about the overall PreLab performance of their groups including highlighting of difficult questions, to be discussed in the subsequent seminar.

The e-laboratories themselves incorporated simulation learning [9] using simulation programs *SimNeuron*, *SimNerv,* and *SimMuscle* from “Virtual Physiology” (http://www.virtual-physiology.com/), teaching videos describing experimental set-ups for EEG, EMG, EOG, as well as hands-on experiments to be conducted at home. The latter included computer-aided experiments for determining the individual hearing ability and visual acuity; two-point discrimination tests; experiments on temperature, taste, and smell sensation as well as sensory and short-term memory. The EEG, EMG, and EOG measurements were conducted on-campus by tutors using the Lt Platform. The students received the data and used the platform to analyze these data and to summarize the results in tables and diagrams. The results and related theoretical and practical issues were discussed during the accompanying organ centered seminars, held online.

The inverted (or flipped) classroom model (ICM) is a well-known teaching concept in which a self-directed learning phase (individual phase) precedes the classroom-instruction phase. In this format, the students accomplish lower-order cognitive processes, like the acquisition and comprehension of knowledge independently, before classroom instruction. The classroom time is subsequently used to execute higher cognitive learning processes, like analysis, synthesis, and evaluation [11, 12]. In our concept, the ICM was the final teaching block, used to recapitulate and discuss the factual/theoretical knowledge obtained previously in asynchronous formats (i.e., from online lectures, textbooks or, in rare cases, provided scientific articles and scripts), to answer all remaining student’s questions and to assure the deep comprehension of the provided material. To this end, we divided the curricular knowledge of neurophysiology into 29 topics, containing 5-7 slides each. The topics not included in the practical course (e.g., cerebellum, basal ganglia, higher cortical functions, etc.), were given more space, whereas those dealt with during the practical course and organ centered seminars were combined but still included for completeness. Each student had to choose his favorite topic at the beginning of the teaching block and present it in a 15 min talk (plus 5 min for discussion) during one of the 5 weekly seminars. The use of provided slides (in a student’s favorite order) was compulsory, but students could add 1-2 slides of their own choice. They were also requested to provide a take-home message in form of 3 main questions posed before the talk and to be answered afterward by fellow students. Questions from the audience as well as the “must know” instructor’s questions were discussed after each talk. The seminars took place via Zoom with talks presented in the shared screen modus.

In an accompanying anonymized study, this e-learning concept was examined for its practicability, effectiveness, and acceptance by the students.

### Experience with the all-digital training course in neurophysiology

Below we briefly describe students’ feedback concerning each building block, as deduced from the second questionnaire.

### Lectures

The students were rather satisfied with the offered online lectures in terms of content relevance, technical performance, and organization, giving these aspects the school grades 2.0, 2.1, and 1.9, respectively. Nearly 75% of the participants also appreciated the availability of lecture scripts, containing all figures used during the lecture. The free-text comments ascertained that the figures provided a clear and detailed overview of the material as well as visual input helping to integrate the content. According to students, the figures were actively used already during the lecture to make notes and for intensified learning before exams. Only three students did not use the lecture scripts because of technical problems or lack of interest.

**Fig. 2:**
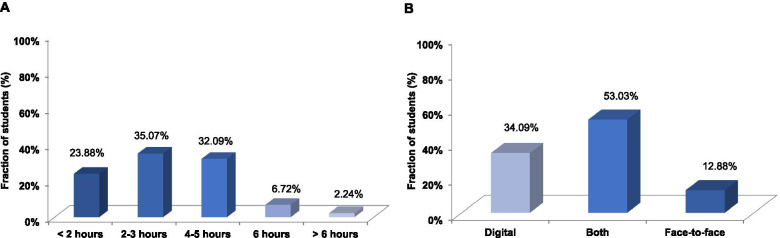
Students’ feedback about online lectures. The daily time spent dealing with lectures (A) and the preferred lecture style (**B**)

To view the lectures, most students used either a laptop (54%) or a tablet (31%), while locally fixed personal computers (7.4%) and smartphones (7.4%) played a minor role. About 67% of students spent 2 to 5 h per day watching and reworking on the lectures, including about 1 h streaming time (Fig. 2A). 24% of students invested less than 2 h. 91% of the latter students explained the reduced time effort as follows: some postponed intense learning to the time immediately before the exam; some had to perform exams in other disciplines, originally scheduled at the beginning of the semester but now taking place during the block of neurophysiology; some preferred to learn from textbooks or were interested solely in topics directly relevant to the exam (for these students the lectures were much too detailed), while others watched lectures as a summary after having learned the topic on their own.

Been asked whether they would prefer digital or face-to-face lectures in the future, 53% of students preferred to have both in parallel (Fig. 2B). For that 34% of students who preferred the digital format, the major arguments were the high flexibility (i.e., watching the lecture whenever it fits best, working through at their own pace), and the time saving for travel to the campus. Likewise, students liked the possibility to stop the lecture at any time either for taking notes, looking up a specific topic in textbooks for clarification/evaluation, revisiting a part of the lecture that was confusing or too fast for understanding, or simply for relaxing (“one is more independent in terms of time, can learn more effectively, looks up questions directly, is not easily distracted, can adjust the speed of the lecture and, if necessary, skip things or look at them again”). A better concentration ability, the equality of opportunities, and better childcare alongside studies were also mentioned (“Better compatible with my everyday life (child and job alongside studies). More equal opportunities, more time for studying”). For that 13% of students who preferred face-to-face lecturing, the main reason was the personal contacts to their fellow students and the lecturers. Also, the ability to directly ask questions or discuss topics with either the lecturer or their classmates was important. Some students missed the strict time frame and the structural organization by a defined time schedule.

Finally, students were requested to suggest constructive improvements to the digital lecture format. The suggestions included technical issues like the tone quality; breakdown of the internet platform, most likely due to internet problems at the student’s home; difficulties to play lectures, including the full-screen modus, on some equipment. The latter problems occurred more often with Apple devices. Here, the future digitalization strategy must consider unified technical standards for the equipment for lecturers and students. Despite the flexibility of e-learning, single lectures were seen as entities. Thus, the wish of many students was to restrict single lectures to a maximum of 45 min by condensing/shortening the lecture or giving a second lecture on the same topic. Some students asked for highlighting the exam-relevant messages even more explicitly. The others wished to make the lectures permanently available or to provide a download format. Several students wished to have an online forum for their questions.

### Practical course

The study participants scored the layout of the Lt Platform as user-friendly, intuitive, and well-organized (1.7 on the school grades scale). The overall content of e-laboratories scored 2.2. The feedback comments praised the useful pop-ups giving background information and explaining the terms used, step-by-step explanations of individual experiments as well as informative figures and schemes. According to the students, all information, required for working with the Lt Platform, was provided in our introduction video, which they could look up at any time. Our students also enjoyed the PreLab tests, especially the “check answer” function, enabling immediate feedback and the variety of different question types, motivating them to understand the task before starting the experiment (“Everything clearly explained and structured, the small tests were great!”). However, when asked whether the interactive online environment was better for learning compared to a regular textbook, 44% of students rated both settings equally well: 32% learned better in the interactive environment, while 24% preferred textbooks. Those, who preferred the interactive environment, justified it by the necessity to “actively” find out the answers instead of “passively” reading books, by well-portioned knowledge “milestones” and by a more satisfying learning experience. The ones, who learned less well in the interactive environment, stressed the lack of motivation for experimental work, their hesitance to learn with online formats, and the feeling to invest more time than necessary (“Some laboratories were too long. I admit, I wanted to finish them quickly. Often I wasn't far enough with my theoretical knowledge and the laboratory was out of sequence for my learning progress”).

In terms of workload and study attitude, the students worked on average 3.78 h per week, 51% of students conducted the online experiments in small learning groups (2-5 people) while 49% worked alone. Asked to suggest constructive improvements for the practical course, the students named either technical issues (e.g., compatibility of the PC operating system and the simulation software) or technical abilities of students (e.g., working with oscilloscopes, installing relevant software), which should be given more considerations. Many participants stressed that practical courses should take place on campus, with tutors and fellow students, who can also serve as subjects for practicing medical examination, and asked to provide the protocols with “correct data” for each e-laboratory. Others again mentioned that practical courses are not the most time-efficient way to obtain knowledge, not recognizing the need for acquiring technical skills.

### Organ centered seminar

These seminars aimed to discuss the theoretical background and the data obtained during the respective e-laboratory, thus assuring the proper knowledge of this specific topic. Usually performed as a dialog between the instructor and the students in a group of 25 students, in summer semester 2020 they took place as synchronous online seminars. In this format, instructors repeatedly reported the difficulty to involve all students in the discussion. The students, however, found the interaction with instructors sufficient (99% of participants) and denied a need for further interactions (84%). In one seminar we further divided the students into 6 teams, placed into 6 breakout rooms. Each team was responsible for one subtopic, which they had to prepare for general discussion. 41% of study participants liked this new seminar structure. The average mark in school grades was 2.8. Those, who were positive about the teamwork experiment, reported that they worked out their “own” topic deeper, were much more attentive, analyzed the data in more detail, discussed the missing links within the group, and “were forced” to talk about their topic in front of other fellow students (“one has actively participated and was more attentive”). Those, who were rather skeptical, reported some technical difficulties when assigning the teams to individual breakout rooms, shortage of time for in-depth discussion, poor interaction between team members as well as difficulties to communicate via Zoom (“Group work during online Zoom meetings is feasible, but very cumbersome and usually does not create a balanced group dynamic. Such online group work does not benefit the learning success”). In total, 46% of participants thought that teamwork helped them to understand the topic better, whereas 54% had an opposite opinion. Following improvements of this seminar format were suggested by the students: discuss the results of individual experiments in more detail and extend the teamwork, attending more to the interaction within the group.

### Integrated seminar using *inverted classroom* concept

When asked whether the ICM helped them to better understand the thematic content of seminars, 52% of the participants acknowledged the inverted classroom as very helpful (school grades 1 and 2), while 14% denied having benefitted from it (school grades 4 and 5). Accordingly, in free-text comments, many supportive and even enthusiastic statements have been received. The students stressed the possibility of peer teaching, helping to identify and address the questions, which may escape the instructor’s notice, and enjoyed more simple and understandable explanations of their peers, often presenting a different view on the problem. Likewise, preparing their own talks helped the students to develop presentation skills and to analyze the topics in more detail (“I understood the explanations of my peers very well. Preparing my own talk helped me to understand the topic in detail”). Many students stressed the necessity to acquire factual knowledge before the classroom phase, thus approving the general ICM concept [11, 12]. Those students, who did not like the ICM, acknowledged benefiting from preparing their own topic but had difficulties to concentrate during presentations of their peers. They perceived this format as instructor-centered but with the peer in the role of instructor and suggested that each student should present one topic per week.

On average, the study participants have spent around 6.6 h to prepare the 15-min-long talk, with 46% of students spending less than 5 and 30% spending between 5 and 10 h. We also asked which materials students used to prepare their talks. The offered choices were: provided slides, textbooks, online platforms, and others; the answer allowed multiple choices. The most popular were textbooks (41% of votes), followed by online platforms (28%), slides only (17% of votes), and others (12%, detailed as lectures, “YouTube” videos, discussions with fellow students, and scientific publications). Asked about the supervision quality during the seminars, the students graded different topic blocks slightly differently, with the average grade fluctuating between 1.0 and 2.2 (mean: 1.8). Rating of the ICM concept in general, revealed that most students favored this format (average school grade 2.1). In free-text comments students reported to become an expert in their own topic; to re-evaluate and re-interpret the knowledge obtained during lectures; to re-focus on most important issues and to have their peers helping them to gain a deeper knowledge about the subject. According to the students, this concise reinforcement, focused and commented on by the instructors, helped to prepare for the exam better and in a more efficient way. The format also helped them to recognize knowledge gaps or inconsistencies (“It was great to notice how you become an expert on a topic yourself. In addition, instructors gave great support… It was a very pleasant situation: you were sometimes more in conversation with each other and learned that even the instructors do not always know everything, because science itself does not yet know everything so exactly”).

Constructive criticism on ICM mostly focused on the gradual loss of concentration during the seminars. Students suggested reducing the seminar’s length either by limiting the number of talks, taking breaks after each talk or by reducing the time for each talk. The others asked instructors to be more critical about the style, clarity, and rhetoric of students’ presentations; to animate more students to participate in the discussion, or suggested allowing more slides to be selected by the students. Many participants complained about the redundancy, stating that this material was already covered by the lectures, thus obviously misunderstanding the general purpose and the methodology of ICM. Finally, many students argued in favor of an intensified general discussion at the end of each talk and suggested that the instructor should ask questions to everyone in the audience, not only to a student giving the talk, and to better elaborate take-home messages.

### Summative evaluation at the end of the online teaching module

To assess the quality of the online teaching in the summer semester 2020 we compared the results of the summative examination (final written exam in either neurophysiology or physiology in general) between our 425 students and students of the previous, face-to-face instructed semesters 2019 and 2018.

**Fig. 3:**
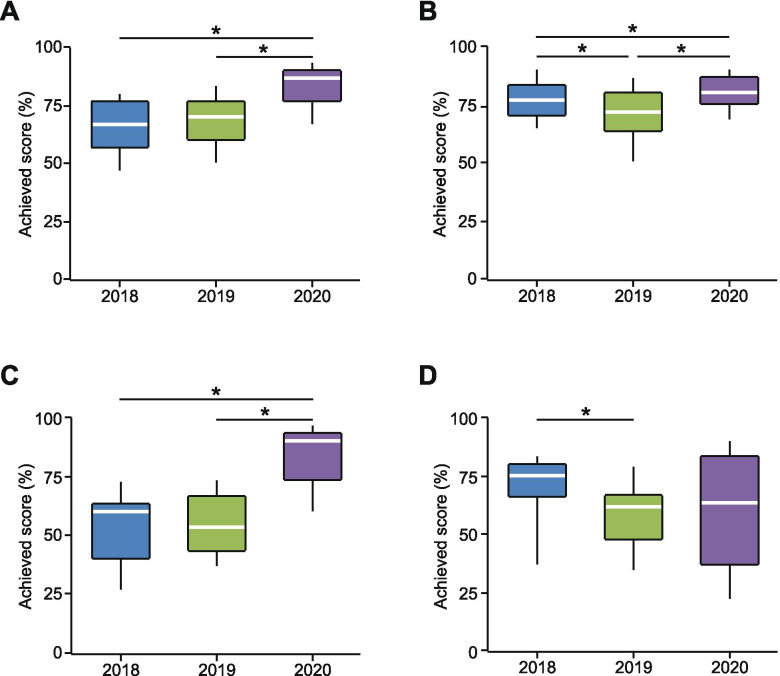
Cohort-specific comparison of the student's performance in the final written exam. Box-and-whisker plots showing the distribution of scores achieved in the final written exam among 3 cohorts of medical students (**A**, 3^rd^ semester; **B**, 4^th^ semester), dentists (**C**), and students of B.Sc. Molecular medicine (**D**). The year of the written exam is shown on the X-axis. For the 3^rd^ semester medical students n= 159, 141, and 137, for 2020, 2019, and 2018, respectively. These students as well as dentists (n = 53, 60, and 41 students in years 2020, 2019, and 2018, respectively) and students of B.Sc. Molecular medicine (n = 17, 32, and 18 students in the years 2020, 2019, and 2018, respectively) wrote the final exam in neurophysiology including 30 single choice questions, in which a maximum of 30 points can be obtained. The 4^th^-semester medical students (n = 161, 151, and 146 students in years 2020, 2019, and 2018, respectively), wrote the final exam in physiology including 60 single choice questions, in which a maximum of 60 points can be obtained. *P ≤ 0.01

As shown in Fig. 3, none of the student cohorts, taught in summer semester 2020, performed worse than their face-to-face instructed peers. In fact, 3 out of 4 cohorts performed significantly better, with students of dentistry showing the most impressive improvement. The performance of both cohorts of medical students and dentists was quite homogeneous. However, the students of B.Sc. Molecular medicine showed extremely heterogeneous results.

After our final written exam in physiology, the students of the 4^th^ semester had to pass the first state exam, consisting of a written and an oral exam. The written part of this examination takes place simultaneously all over Germany, with medical students from 29 German universities participating in the exam. In the physiology part of this examination, our students reached rank 2 in 2018 (81.9% of all questions answered correctly), rank 1 in 2019 (80.9% of correct answers), and rank 3 in 2020 (74.5% of correct answers).

### Overall evaluation of the online teaching module in neurophysiology

During the module, the students on average have spent some 28 h/week studying neurophysiology, with 22.7% of the participants investing 10-20 h, 38.3% investing 20-30 h, and 10.2% investing more than 40 h. 56.3% of the participants felt that the time needed was just right, while 38.3% considered the module as being too time-consuming. In free-text comments, the latter complained about technical difficulties to find the information needed without the library, the need to accomplish the practical lessons themselves instead of doing them in a group, and profiting from the knowledge of their peers. Some students also wished to reduce the content of the module only to those topics, which they felt would be important for the subsequent state exam. Asked whether they had any technical difficulties participating in the synchronous lessons (seminars) 35.9% of participants answered “never”, 42.2% answered “seldom”, 18.0% answered “from time to time” and only 3.9% had frequent technical problems. The main technical problems they experienced included bad quality of the internet connection (n=34) as well as technical problems with macOS (the incompatibility with some simulation programs and the dissociation of film and tone of the lectures, when played on iPad). Asked explicitly about the two main technical platforms used (Lt Platform and ILIAS), the students reported having very few technical problems when using these platforms. ILIAS, however, apparently had some performance problems during the morning hours, when most students wanted to watch the lectures. Some students reported difficulties viewing the sample data on Lt, especially if the internet connection was unstable.

Besides the technical aspects, the main problems, experienced by the students during the e-learning, included the need and the corresponding difficulty to develop own learning schedule and to stay motivated and disciplined, the passive participation and the difficulty to concentrate during the synchronous seminars, the lack of feedback about their learning performance and that of the fellow students, the lack of communication with tutors and peers, spending too much time in front of the computer screen, the lack of good learning conditions, the lack of alternation, all days appear similar to each other, the lack of practical training, inefficient time-management, and the lack of feedback from the audience during own presentations. Consistently, the literature data suggest that computer-aided communication is less effective than face-to-face interaction [5, 13].

Asked whether the combination of the synchronous and asynchronous formats helped them to learn better, 73.4% responded with yes, 22.7 % were undecided and 3.9% of students said no. The free text comments mostly prized the ICM concept in combination with lectures introducing the topic, allowing students to work it out and reflect at their own pace. Regarding seminars, students liked to discuss more difficult and unclear issues within the group, clarify the open questions, and evaluate the learning success. Those students, who were studying general and exercise physiology in the face-to-face format in winter semester 2019/2020, were asked to compare their learning experiences in both courses. Out of 68 participants, 79.4% responded that their experience was comparable or better during the online semester, 20.6 % reported that their learning experience was worse. The reasons, extracted from free-text comments, pointed in the first place to the different format of the practical course. While in summer 2020 each student had to conduct online experiments, in the face-to-face teaching block the experiments were conducted by groups of 5-6 students. Those, who preferred the online training, reported that conducting each experiment alone is much more profitable for understanding the subject and that they were able to devote more time to interesting questions. The newly developed PreLabs also helped them to understand the topic better. Those, who liked face-to-face training more, pointed to the regular structure of the teaching block, helping them to be more disciplined, to procrastinate less, and to actively work through the lectures to be able to follow the lecture the next day.

Finally, the students were asked about their overall opinion about the quality of the online neurophysiology teaching module. The average mark in school grades was 1.9. The students identified the online lectures and the PreLabs as building blocks with substantial added value, to be kept after the COVID-19 pandemic. Despite positive remarks about the e-laboratories listed above, the majority of students voted for face-to-face practical training.

## Discussion

Like all universities in Germany and most universities worldwide [10], we had to switch from face-to-face to digital training in practically no time. According to the literature, however, typical planning, preparation, and development time for an online course is 6-9 months and faculty usually become comfortable with teaching online by the second or third iteration of their courses [14]. The current study allowed us to learn how this switch impacted our students and how the gained experience contributes to best practices of online teaching and learning [15]. To our knowledge, this is the first study describing and analyzing the complete online training course in neurophysiology, including all the aforementioned building blocks. The results show a general acceptance of the newly developed e-learning concept. The quality of the concept is also reflected by the results of the written exam in neurophysiology. Here the results were at least equal to the results of the two prior years, utilizing face-to-face teaching (Fig. 3). The solid knowledge of our 4^th^-semester medical students was further confirmed by the results of the written part of the Germany-wide first state exam, in which they reached rank 3 out of 29.

While recent systematic reviews also suggested that offline and online teaching are equivalent in terms of factual knowledge and examination outcomes [16, 17], practical skills remain a pertinent barrier to online teaching of medical students. Consistently, our students also stressed the importance of the live practical experience for obtaining relevant skills. While many universities restricted their online physiology courses to educational videos and case studies [15, 18, 19], we simulated the complete laboratory environment. From this point of view, our study complements a recent international study, describing the use of similar approaches and virtual tools, and providing educators’ views on the transition to remote physiology laboratories [20]. Ten educators from Australia, Canada, the U.K., and the U.S.A. [20] and 146 students from Germany (our study) agree that virtual laboratories are a good supplement, but not a replacement for the on-campus laboratories; appreciate the value of the asynchronous PreLabs as well as difficulties to transfer skills around teamwork (collaboration and cooperation with others, valuing different views and communicating effectively) and the physiology-related professional skills. Still, the e-laboratories received “good” as an overall mark, proving a valid substitution for on-campus teaching if the pandemic holds on. In its current form, our e-laboratories combine hands-on experiments to be conducted at home (regarding taste, smell, skin somatosensation) with simulation- (working with isolated nerves and muscles) and computer-aided (e.g., measuring hearing ability and visual acuity) experiments. Supplemented by the accompanying PreLabs, further digital tools like the ones developed by OpenSim (https://opensim.stanford.edu) or BodyWorks [21] and by the scalable formative assessment tools (also in form of group competitions or games (e.g., Kahoot!) [15, 22]), this interactive online format will help to teach students in the following years. Further development of this teaching concept in the future might benefit from the multidimensional virtual reality approach providing the students realistic visual, acoustic, and maybe even haptic experiences [23]. It is also worth providing an online chat portal, enabling educators to respond quickly to anonymous student questions [18].

Despite the encouraging results described above, our study revealed several general issues to be addressed before the further extensive use of online formats. One problem regarding online teaching is the internet availability and stability as well as the technical equipment of the participants. Especially for synchronous events, a stable internet connection is an inevitable prerequisite. Likewise, inappropriate technical equipment of some students should be taken into account. It is difficult to imagine that using smartphones more than a very imprecise impression (e.g., of digital lectures with all the complex figures and graphs) could be obtained. This may also apply to some rather small and technically imperfect tablets, raising the question of whether advanced digital learning needs access to high-quality end devices for all students. Thus, it would be advisable to develop course-specific recommendations concerning the quality of end devices, as the students, when working on their own, may not be able to appreciate the loss of quality. The limited compatibility of some programs with Microsoft or macOS operating systems should also be considered. Many students seemed overstrained by the need to install the simulation programs, especially when compatibility problems occurred. Thus, the basic knowledge of IT among the students as well as adequate internet facilities are prerequisites for successful online teaching [24]. Likewise, not all students are equally well trained to work with basic laboratory equipment like oscilloscopes, electrical stimulation devices, etc. This issue is less important when working in groups of 6 students under the guidance of a tutor but becomes essential when working individually in an online format.

Other issues, addressed by our students and also noticed by other educators teaching through the COVID-19 pandemic using remote platforms, are the need to create organization and structure to support learning and the importance of the learner’s engagement [10, 20, 24, 25]. In contrast to traditional face-to-face teaching, where information mostly flows from the teacher to the students, the online formats are more student-centric. This includes all mentioned above advantages (e.g., flexibility in terms of time and place, studying at the own pace, better equality of learning opportunities, saving time on traveling) but also disadvantages (higher need for motivation, self-discipline, and effective time management; in-depth understanding may be difficult; social isolation and lack of help when technical problems occur) [16, 24]. In synchronous formats, learner engagement can be promoted using online chat features, electronic hand-raising for questions, online polling, or the use of breakout rooms enabling students to work in teams [10, 11, 16, 26]. While only half (46%) of our students have found the breakout room strategy helpful, the improvements, suggested by the students (increase the time of the breakout session, discuss the results in more detail) might improve the acceptance of this format in the future.

The value of time was another important issue raised by the study. Obviously, many study participants were prepared to invest time into the knowledge-delivering aspect of preclinical training, largely disregarding the value of learned practical skills. There are several possible explanations for this finding: most of our students just finished school, which makes their study attitude more school-like; the students are learning for the first state exam, traditionally emphasizing theoretical knowledge. So, their understanding of “what is important for the exam” is based on the analyses of questions of previous written exams. This underestimates the recent trend towards the skills-oriented curriculum [27–29]. The devices used (or simulated) during the practical course are technologically less advanced and might be more difficult to operate than those currently used in clinics. For example, the frequent lack of in-depth school training in physics makes the use of basic electrophysiological equipment difficult. Thus, both the modernization of the laboratory equipment and a better explanation of what and why is important to learn, are essential.

Consistent with the above-mentioned value of theoretical knowledge, the online lectures were used by many participants not only as the introduction to a given topic but also for preparing for the seminars, practical courses, their own ICM talks, and exams. Interestingly, when asked which lecture format they do prefer for the future, most students voted for both, digital and face-to-face lectures. In such a double format a face-to-face lecture can be used for answering difficult questions and an e-lecture - for basic knowledge acquisition before the face-to-face lecture or consultation when learning for exams. At first glance, this statement conflicts with the above-mentioned value of time, so pronounced among the students. It reveals, however, a problem, often ignored during face-to-face lecturing, that students possess different learning abilities and background knowledge [4]. An e-lecture covering the basic facts including options for fast forward, pause, and rewind as well as an online chat portal would offer possibilities for all students to acquire necessary knowledge at their own learning pace and would help to equalize their expertise. From here, a face-to-face lecture dealing with more sophisticated contents of the topic could be much more focused and would reach all or at least most students alike. The e-lecture would thus replace the textbook-oriented self-study of the basics prior to the classical face-to-face lecture. It seems, however, not far-fetched to assume that traditional textbooks will also be transformed to online formats in the nearest future. This would provide publishers with easy access to the content for correcting faults or misleading statements and for updating the content continuously. An e-lecture, including questions in the way described here for the PreLabs, could indeed increase the motivation and discipline of all students, and strengthen/equalize their background knowledge.

The inverted classroom model was also the format students appreciated. In e-learning settings, this format was ideally suited to actively involve students in and to promote the individualization of the learning process [12]. ICM stimulates the self-motivated study of a given topic, consideration of how it fits into the overall thematic context, and oral presentation of the results either face-to-face or -as in this case- digitally. It is the next logical student-centered learning step, following the e-lecture. It gets students from a mere recipient of learning contents to somebody who compiles and reflects information to tell a comprehensive story and to defend it in a lively discussion. Therefore, ICM is also particularly suited for identifying knowledge gaps that were left undetected during conventional lecturing [11, 26].

Finally, many students were missing chat rooms to present their questions anonymously, as they felt timid to ask questions during an online meeting. This reflects the experience of many lecturers worldwide [16, 18]. Although students should be stimulated and supported to build up enough self-confidence to present statements and questions openly, there is no reason to deny the use of a chat room, especially as many questions may appear at the time when the synchronous lecture/seminar is over.

## Conclusions

No question, the necessity to develop online teaching formats *stante pede* was a challenging and demanding process, leaving room for improvement. Still, our goal was to create a comprehensive high-quality online training course in neurophysiology. According to students’ surveys, the longitudinal internal assessment (Fig. 3), and the summative comparison across the medical schools in Germany (see above), this course proofed to be of good educational value and at least as effective for content knowledge as our pre-COVID on-campus course. For learning practical skills, the virtual laboratories turned out to be a good supplement, but not a replacement for the on-campus laboratories. However, some newly developed e-learning blocks (e.g., interactive PreLabs or digital lectures) have a substantial added value worth transferring into the post-COVID-19 era, thus contributing to the best practices of online teaching and learning. In the long term, such online courses should equip students with valuable experience for the anticipated shift in medical practice towards virtual medicine.

## Data Availability

The datasets used and/or analyzed during the current study are available from the corresponding author on reasonable request.

## Abbreviations

EEG: electroencephalography
EMG: electromyography
EOG: electrooculography
a.h.: academic hours
ICM: inverted classroom model

## References

[1] Krishnan A, Hamilton JP, Alqahtani SA, Woreta TA: A narrative review of coronavirus disease 2019 (COVID-19): clinical, epidemiological characteristics, and systemic manifestations. *Intern Emerg Med* 2021. 10.1007/s11739-020-02616-5 PMC7811158 33453010

[2] Elmer SJ, Durocher JJ (2020). Moving student research forward during the COVID-19 pandemic. Adv Physiol Educ.

[3] Gill D, Whitehead C, Wondimagegn D (2020). Challenges to medical education at a time of physical distancing. Lancet.

[4] Kachra R, Brown A (2020). The new normal: Medical education during and beyond the COVID-19 pandemic. Can Med Educ J.

[5] Kaur S, Bir M, Chandran DS, Deepak KK (2021). Adaptive strategies to conduct participant-centric structured virtual group discussions for postgraduate students in the wake of the COVID-19 pandemic. Adv Physiol Educ.

[6] Clarke V, Braun V (2013). Teaching thematic analysis: Overcoming challenges and developing strategies for effective learning. The Psychologist.

[7] Tie YC, Birks M, Francis K (2019). Grounded theory research: A design framework for novice researchers.

[8] Watling CJ, Lingard L: Grounded theory in medical education research: AMEE Guide No. 70. *Med Teach* 2012, 34(10):850-861. 10.3109/0142159X.2012.704439 22913519

[9] Hall AK, Nousiainen MT, Campisi P, Dagnone JD, Frank JR, Kroeker KI, Brzezina S, Purdy E, Oswald A (2020). Training disrupted: Practical tips for supporting competency-based medical education during the COVID-19 pandemic. Med Teach.

[10] Gordon M, Patricio M, Horne L, Muston A, Alston SR, Pammi M, Thammasitboon S, Park S, Pawlikowska T, Rees EL (2020). Developments in medical education in response to the COVID-19 pandemic: A rapid BEME systematic review: BEME Guide No. 63. Med Teach.

[11] Tolks D, Schafer C, Raupach T, Kruse L, Sarikas A, Gerhardt-Szep S, Kllauer G, Lemos M, Fischer MR, Eichner B *et al*: An Introduction to the Inverted/Flipped Classroom Model in Education and Advanced Training in Medicine and in the Healthcare Professions*. GMS. J Med Educ* 2016, 33(3):Doc46. 10.3205/zma001045 PMC4894356 27275511

[12] Wannemacher K, Jungermann I, Scholz J, Tercanli H, von Villiez A: Digitale Lernszenarien im Hochschulbereich. *Hochschulforum Digitalisierung* 2016: 1-113.

[13] Warkentin ME, Sayeed L, Hightower R (1997). Virtual teams versus face-to-face teams: an exploratory study of a web-based conference system. Decis Sci.

[14] Hodges C, Moore S, Lockee B, Trust T, Bond A: The difference between emergency remote teaching and online learning. . *EDUCAUSE Review* 2020.

[15] Davis CP, Pinedo T. The Challenges of Teaching Anatomy and Physiology Laboratory Online in the Time of COVID-19. *J Microbiol Biol Educ*. 2021;22(1). 10.1128/jmbe.v22i1.2605 PMC8011876 33884057

[16] Dost S, Hossain A, Shehab M, Abdelwahed A, Al-Nusair L (2020). Perceptions of medical students towards online teaching during the COVID-19 pandemic: a national cross-sectional survey of 2721 UK medical students. BMJ Open.

[17] Saiyad S, Virk A, Mahajan R, Singh T (2020). Online Teaching in Medical Training: Establishing Good Online Teaching Practices from Cumulative Experience. Int J Appl Basic Med Res.

[18] Casotti G, Beneski JT, Knabb MT (2013). Teaching physiology online: successful use of case studies in a graduate course. Adv Physiol Educ.

[19] Mutch-Jones K, Sengupta N, Minor VC, Goudsouzian LK (2021). Professional science education videos improve student performance in nonmajor and intermediate biology laboratory courses. Biochem Mol Biol Educ.

[20] Choate J, Aguilar-Roca N, Beckett E, Etherington S, French M, Gaganis V, Haigh C, Scott D, Sweeney T, Zubek J (2021). International educators' attitudes, experiences, and recommendations after an abrupt transition to remote physiology laboratories. Adv Physiol Educ.

[21] Martay JLB, Martay H, Carpes FP (2021). BodyWorks: interactive interdisciplinary online teaching tools for biomechanics and physiology teaching. Adv Physiol Educ.

[22] Moro C, Phelps C, Stromberga Z (2020). Utilizing serious games for physiology and anatomy learning and revision. Adv Physiol Educ.

[23] Singh RP, Javaid M, Kataria R, Tyagi M, Haleem A, Suman R (2020). Significant applications of virtual reality for COVID-19 pandemic. Diabetes Metab Syndr.

[24] Deepika V, Soundariya K, Karthikeyan K, Kalaiselvan G: 'Learning from home': role of e-learning methodologies and tools during novel coronavirus pandemic outbreak. *Postgrad Med J* 2020. 10.1136/postgradmedj-2020-137989 PMC10016931 33154099

[25] Ahmed S, Shehata M, Hassanien M (2020). Emerging faculty needs for enhancing student engagement on a virtual platform. MedEdPublish.

[26] Tolks D, Romeike B.J.E, Kuhn S, Kleinsorgen C, Huber J, Fischer M, Bohne C, Hege I: The online inverted classroom model (oICM). A blueprint to adapt the inverted classroom to an online learning setting in medical and health education. . *MedEdPublish* 2020. 10.15694/mep.2020.000113.2 PMC10702666 38073851

[27] Frank JR, Snell LS, Cate OT, Holmboe ES, Carraccio C, Swing SR, Harris P, Glasgow NJ, Campbell C, Dath D (2010). Competency-based medical education: theory to practice. Med Teach.

[28] Frenk J, Chen L, Bhutta ZA, Cohen J, Crisp N, Evans T, Fineberg H, Garcia P, Ke Y, Kelley P (2010). Health professionals for a new century: transforming education to strengthen health systems in an interdependent world. Lancet.

[29] Frank JR, Snell L, Englander R, Holmboe ES, Collaborators I (2017). Implementing competency-based medical education: Moving forward. Med Teach.

